# Association between axial length and perfluoropropane gas duration after pars plana vitrectomy with fixed-volume pure gas injection

**DOI:** 10.5935/0004-2749.2025-0238

**Published:** 2025-11-04

**Authors:** Rodrigo Pessoa Cavalcanti Lira, Ana Paula Teles Silveira, Gabriel Rocha Lira, Maria Isabel Lynch Gaete

**Affiliations:** 1 Universidade Federal de Pernambuco, Recife, PE, Brazil

Dear Editor,

The duration of perfluoropropane (C_3_F_8_) gas tamponade after pars plana vitrectomy (PPV) is a critical determinant of surgical outcomes but varies considerably among patients. Accurate prediction of gas duration is essential for postoperative planning and patient counseling. Most published studies have used prediluted gas at isovolumetric concentrations^(^1^-^4^)^. In this study, we examined the association between axial length (AL) and C_3_F_8_ gas duration using an alternative method: injection of a fixed volume of pure (100%) gas.

We conducted a retrospective case series at a single center, including 66 eyes of 66 pseudophakic patients who underwent 25-gauge PPV by a single surgeon between 2021 and 2024. Eligible patients were aged ≥50 year, with no prior vitreoretinal surgery. Those with glaucoma, retinal detachment, or abnormal globe morphology were excluded. The surgical protocol was standardized: 25-gauge vitrectomy, fluid–air exchange, and transscleral injection of 0.7 mL of 100% C_3_F_8_, with passive drainage of excess gas. The primary outcome was gas duration (days), as reported by patients; secondary outcomes included changes in intraocular pressure (IOP). Correlations were analyzed using Pearson or Spearman coefficients, and a simple linear regression model was constructed. Statistical significance was set at *p*<0.05. The study complied with the Declaration of Helsinki.

The cohort (50% women) had a mean (standard deviation [*SD*]) age of 67.5 (7.9) year. Median AL was 24.49-mm (interquartile range, 23.77–27.24-mm), and mean gas duration was 35.6 (3.9) days. A strong negative linear correlation was observed between AL and gas duration (Pearson r=−0.85; 95%CI, −0.91 to −0.76; p<0.001) (Figure 1). AL alone explained 72.3% of the variability in gas duration (R²=0.723). The predictive equation was gas duration (days)=69.14−1.33×AL (mm).

**Figure 1:**
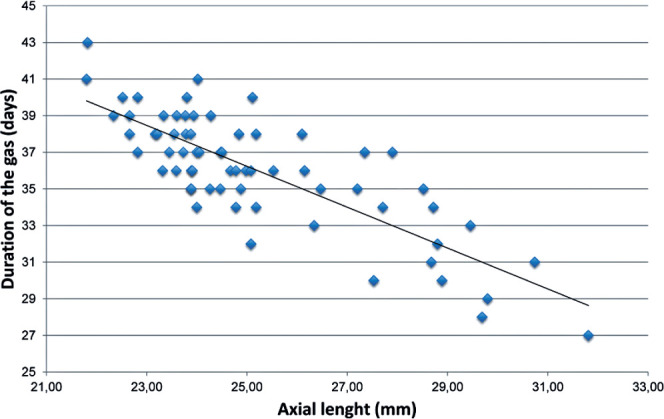
Scatter plot of C_3_F_8_ gas duration versus axial length.

Mean (SD) IOP increased from 14.1 (2.2) mmHg preoperatively to 14.9 (3.1) mm Hg on postoperative day 1 (mean difference, 0.79-mm Hg; p=0.015), returning to baseline by Week 26 (14.4 mmHg; p=0.230 vs. baseline). AL showed a moderate negative correlation with the Day 1 IOP change (Spearman ρ=−0.41; p=0.001), suggesting that shorter eyes experienced greater acute IOP elevations.

In this study, AL emerged as a robust predictor of intraocular C_3_F_8_ gas duration. This association likely reflects our technique of injecting a fixed volume of pure gas. Unlike prediluted gas methods that produce a uniform final concentration, our approach yields a final concentration inversely proportional to vitreous cavity volume, for which AL is a reliable surrogate^(^1^,^2^,^4^)^.

Faster gas absorption in longer eyes likely results from two mechanisms: (1) a geometric effect, in which a larger vitreous cavity provides greater surface area for diffusion; and (2) a concentration effect, in which dilution within a larger cavity lowers the initial gas concentration, reducing half-life. These mechanisms may explain why studies using pre-diluted gas failed to demonstrate a significant AL–duration correlation^(^2^,^4^)^.

The negative correlation between AL and acute IOP elevation further supports the concentration hypothesis. Shorter eyes, exposed to higher final concentrations, likely experience greater bubble expansion and IOP spikes^(^5^)^.

Study limitations include its retrospective design and reliance on patient-reported gas duration. Nonetheless, the regression model offers a clinically useful tool for tailoring postoperative restrictions and identifying patients at risk for acute ocular hypertension.

In conclusion, AL strongly and inversely correlates with the intraocular duration of C_3_F_8_ gas when injected as a fixed volume of pure gas during vitrectomy. Additionally, shorter eyes are at increased risk of acute postoperative ocular hypertension. A prospective randomized trial is warranted to clarify the relative roles of ocular geometry and gas concentration.

## Data Availability

The datasets generated and/or analyzed during the current study are included in the manuscript.
